# Potential categories of perceived recurrence risk in stroke patients and their relationship with self-management ability

**DOI:** 10.3389/fpubh.2025.1596812

**Published:** 2025-05-16

**Authors:** Yihao Wu, Shitong Gong, Fei Long, Jie Yin

**Affiliations:** ^1^Department of Neurosurgery, Xuzhou Central Hospital, Affiliated Xuzhou Clinical College of Xuzhou Medical University, Xuzhou, Jiangsu, China; ^2^Department of Neurology, Xuzhou Central Hospital, Affiliated Xuzhou Clinical College of Xuzhou Medical University, Xuzhou, Jiangsu, China

**Keywords:** recurrence risk perception, self-management ability, latent profile analysis, influencing factors, correlation

## Abstract

**Objective:**

This study aimed to identify latent profile categories of recurrence risk perception among stroke patients, analyze the factors influencing these categories, and examine their correlation with self-management ability. The findings are intended to provide new insights and a foundation for enhancing self-management in stroke patients.

**Methods:**

A total of 221 stroke patients admitted to the Department of Neurology and Neurosurgery at Xuzhou Central Hospital between January 2024 and December 2024 were selected using a convenience sampling method. Data were collected using a general information questionnaire, the Stroke Patient Recurrence Risk Perception Assessment Scale, and the Stroke Patient Self-Management Ability Assessment Scale. Statistical analyses included factor analysis and pairwise comparisons to examine influencing factors and their relationships.

**Results:**

A total of 221 questionnaires were distributed, with 23 invalid responses excluded, yielding an effective response rate of 89.59%. The mean recurrence risk perception score was 39.63 ± 5.67, while the mean self-management ability score was 85.69 ± 12.33. Latent profile analysis identified three distinct categories of recurrence risk perception: high -level, medium -level, and low -level risk perception groups. Univariate analysis revealed significant differences in recurrence risk perception based on age (*χ*^2^ = 11.132, *p* = 0.025), education level (*χ*^2^ = 5.523, *p* = 0.044), living environment (*χ*^2^ = 9.868, *p* = 0.007), disease duration (*χ*^2^ = 13.142, *p* = 0.011), and stroke frequency (*χ*^2^ = 25.710, *p* < 0.001). Logistic regression analysis indicated that age, living environment, disease duration, and stroke frequency were protective factors for recurrence risk perception (*OR* < 1, *p* < 0.05). Pairwise comparisons showed that patients in the high -level risk perception group exhibited significantly higher self-management ability compared to those in the medium -level (*p* < 0.01) and low -level risk perception groups (*p* < 0.01). Similarly, patients in the medium -level risk perception group demonstrated greater self-management ability than those in the low -level risk perception group (*p* < 0.01).

**Conclusion:**

This study utilized latent profile analysis to classify stroke patients’ recurrence risk perception into three categories: high, medium, and low. These profiles were influenced by age, living environment, disease duration, and stroke frequency. Moreover, recurrence risk perception was significantly associated with self-management ability. Clinical practitioners should focus on patients in the low -level risk perception group, implementing targeted interventions to enhance their awareness of recurrence risk, thereby improving self-management, preventing recurrence, and optimizing patient outcomes.

## Introduction

Stroke has emerged as the leading cause of mortality and disability (1) worldwide. As a chronic, non-communicable neurological disease, stroke (2) poses a significant public health burden, characterized by a prolonged treatment course, rapid disease progression, complex management strategies, and high mortality rates Epidemiological data indicate that the recurrence rate among first-time stroke patients reaches 17.0% within 1 year and 46% within 5 years (3–5). Recurrent stroke significantly increases mortality (6), while exacerbating the decline in patients’ quality of life and imposing a greater caregiving burden (7, 8). Consequently, mitigating stroke recurrence is crucial for reducing morbidity and mortality, enhancing quality of life, and alleviating the socioeconomic burden associated with stroke.

A strong awareness of recurrence risk among stroke patients enables early identification of warning signs, modification of harmful behaviors, promotion of functional recovery, and ultimately, prevention of stroke recurrence (9, 10). However, current research on recurrence risk perception remains limited to cross-sectional analyses of influencing factors (5, 11, 12, 26), lacking a deeper exploration of underlying mechanisms. Self-management ability refers to the comprehensive ability of individuals to consciously plan, regulate, monitor and control their thoughts, emotions and behaviors in many aspects such as cognition, emotion and behavior, so as to achieve personal goals, maintain physical and mental health, adapt to environmental changes and promote their own development. Better self-management ability of stroke patients can better medication adherence, symptom monitoring, and change bad lifestyle, which is crucial to reduce stroke recurrence. The Transtheoretical Model (13) posits that self-management ability directly influences behavioral change. Therefore, assessing stroke patients’ self-management ability and providing targeted interventions for those with lower levels can enhance health management outcomes. The health belief model (HBM) is a theoretical model that uses social psychological methods to explain health-related behaviors (14). As an important part of the health belief model, risk perception is the product of perceived susceptibility and perceived severity, indicating an individual’s cognition of the threat and severity of a certain behavior or disease. Patients’ perception of the risk of stroke recurrence, including the cognition of disease severity and susceptibility, is the core element. When patients perceive that the risk of recurrence is high and the consequences are serious, if they have the right health beliefs, they will have a positive self-management motivation. This motivation encourages the patient to adopt a healthy lifestyle in behavior, such as reasonable diet, regular exercise, and regular medication. At the same time, patients’ self-efficacy also plays a moderating role. High self-efficacy helps to strengthen positive behaviors, while vice versa may hinder behavior change.

In this context, this study applied latent profile analysis (LPA) to identify distinct subgroups of stroke patients based on their recurrence risk perception and to explore the relationship between these subgroups and self-management ability. The findings aim to provide clinical guidance for identifying and supporting patients with low levels of recurrence risk perception, thereby improving overall stroke management.

## Materials and methods

### Clinical data

A total of 221 stroke patients admitted to the Department of Neurology and Neurosurgery at Xuzhou Central Hospital between January to December 2024 were selected using a convenience sampling method.

Inclusion criteria: (1) Patients who met the diagnostic criteria outlined in the Chinese guidelines for the diagnosis and treatment of acute stroke, confirmed by head CT; (2) Aged between 18 and 85 years; and (3) Cognitively intact, with normal verbal communication or reading and writing abilities. Using MMSE assessment scale, score≥24 points.

Exclusion criteria: (1) Presence of organic brain injury or severe organ dysfunction affecting the heart, liver, or kidneys; and (2) Diagnosed psychiatric disorders, including anxiety or other mental health conditions.

Sample size calculation: Based on the method proposed by Ni et al. (15) and Fang et al. (28), the study utilized three survey scales comprising a total of 20 variables. The recommended sample size is 5 to 10 times the number of variables. To account for potential missing or invalid responses, the initial sample size was increased by 20%. The final sample size calculation was as follows: *n* = 20*5/ (1–20%) = 125 ~ 250.

A total of 210 questionnaires were distributed during the actual survey. The study was approved by the Ethics Committee of Xuzhou Central Hospital (Approval No.: 2023-120069).

### Survey tools

#### General information questionnaire

A self-designed questionnaire was used to collect demographic and clinical information, including age, gender, educational level, place of residence, living arrangements, economic status, marital status, medical insurance type, underlying conditions, stroke frequency, and disease duration.

#### Recurrence risk perception assessment scale for stroke patients

The scale, developed by Lin et al. (16) and Snowdon et al. (27), consists of two parts. This study utilized only the second part, which assesses recurrence risk perception across three dimensions: (1) perceived severity of recurrence (7 items), (2) perceived behavioral risk factors for recurrence (6 items), and (3) perceived disease-related risk factors for recurrence (4 items). Responses are scored on a three-point Likert scale (1 = “disagree” to 3 = “agree”), with total scores ranging from 17 to 51. Higher scores indicate a greater perceived risk of stroke recurrence. The total Cronbach’s *α* coefficient, split half reliability and retest reliability were 0.905, 0.763, and 0.897, respectively. In this study, the Cronbach’s *α* coefficient of this scale was 0.824.

#### Stroke self-management ability assessment scale

Originally developed by Boger et al. (17) and later adapted for use in China by Wang et al. (18, 29), this scale measures six dimensions: (1) communication confidence (4 items), (2) professional guidance perception (3 items), (3) self-management decision-making (4 items), (4) stroke impact (4 items), (5) perception confidence (4 items), and (6) health management ability (4 items). The scale includes a total of 23 items, each rated on a six-point Likert scale (1 = “completely incorrect” to 6 = “completely correct”), with total scores ranging from 23 to 138. Higher scores indicate greater self-management ability, promoting adherence to healthy behaviors.

### Research design

This paper is a cross-sectional study.

The dependent variable was the perceived score of recurrence risk, and the independent variable was demographic data, past history information, duration of disease, stroke frequency, etc.

### Data collection

All questionnaires were administered by trained investigators upon patient admission. Before distribution, the study objectives and survey content were explained, and informed consent was obtained. During questionnaire completion, investigators ensured that no items were left unanswered or incorrectly filled. Upon submission, questionnaires were reviewed for completeness and validity, with invalid responses excluded. To ensure data accuracy, survey results were recorded independently by two researchers.

### Statistical analysis

The recovered data met the normal distribution. Statistical analyses were performed using *t*-tests and chi-square tests to evaluate the influence of various factors on different latent profile groups, with a significance level of *p* < 0.05.

When processing missing data, multiple interpolation methods were used to generate multiple complete data sets for analysis and then the results were merged to process the data containing missing values.

Latent profile analysis (LPA) was conducted using Mplus 8.3 to classify stroke patients based on their recurrence risk perception. Model selection was performed by gradually increasing the number of categories and comparing goodness-of-fit indices, including:

Log-likelihood function value (Log(L)).

Akaike information criterion (AIC).

Bayesian information criterion (BIC).

Sample-size adjusted BIC (aBIC).

Lower values of these indices indicate better model fit. Additional model validation was conducted using:

Lo–Mendell–Rubin Adjusted Likelihood Ratio Test (LRT).

Bootstrap Likelihood Ratio Test (BLRT) *p*-values to assess model significance.

Entropy was used to evaluate classification accuracy, with values closer to 1.0 indicating better precision. Pearson correlation analysis was performed to assess relationships between variables.

## Results

### Basic information and recurrence risk perception scores

A total of 221 questionnaires were distributed, with 23 deemed invalid, yielding an effective response rate of 89.59%. Among them, 72 cases (36.36%) of hemorrhagic stroke, 126 cases (63.64%) of ischemic stroke. Recurrence risk perception scores ranged from 22 to 50, with a mean total score of 39.63 ± 5.67 and an average item score of 2.33 ± 0.49. Patient demographic and clinical characteristics are presented in Table 1.

**Table 1:** General information of respondents (*n* = 198).

Items	Categories	*n*	(%)	Items	Categories	*n*	(%)
Age (years)	<45	11	5.56	Marital status	Unmarried	5	2.53
	45 ~ 59	84	42.42		Married	181	91.41
	≥60	103	52.02		Divorced	12	6.06
Gender	Male	105	53.03	Hypertension	Yes	77	38.89
	Female	93	46.97		No	121	61.11
Literacy	Junior and below	94	47.47	Coronary heart disease	Yes	58	29.29
	Senior	78	39.39		No	140	70.71
	College and above	26	13.14	Diabetes	Yes	49	24.75
Place of residence	City	71	35.86		No	149	75.25
	Township	55	27.78	Duration of disease (years)	<1	84	42.42
	Rural	72	36.36		1 ~ 3	66	33.33
Residence style	Living alone	25	12.63		>3	48	24.24
	Family residence	173	87.37	Number of strokes	1	155	78.28
Monthly income (yuan)	<4,000	91	45.96		2	34	17.17
	4,000	68	34.34		≥3	9	4.55
	7,000	27	13.64	Medicare payment method	Resident health insurance	101	51.01
	>10,000	12	6.06		Employee health insurance	97	48.99

### Potential profiling of relapse risk perception in stroke patients

In this study, the researchers utilized the scores from 17 items to assess recurrence risk perception. They initiated the analysis with a baseline model (category number 1) and progressively fitted models ranging from 1 to 4 potential profiles, as detailed in Table 2. Three potential profile models were identified as the optimal model for assessing the recurrence risk perception among stroke patients.

**Table 2:** Comparison of fitting parameter indexes of different potential profile models (*n* = 198).

Models	AIC	BIC	aBIC	Entropy	LRT	BLRT	Category probability
1	10720.406	10799.347	10440.538	1.000			
2	8514.927	8650.631	8514.239	0.935	0.008	<0.001	0.31/0.69
3	7322.546	7515.007	7331.025	0.958	0.011	<0.001	0.21/0.36/0.43
4	6848.141	7097.360	6865.819	0.981	0.301	<0.001	0.24/0.11/0.20/0.45

### Mean attribution rates for three potential categories of perceived risk of stroke recurrence among patients

To assess the reliability of the analysis results for the aforementioned potential profile categories, the average attribution probability for the three categories within each sample was computed. The findings indicate that the probability of accurate classification for potential class 1 is 98.0%, for potential class 2 is 98.2%, and for potential class 3 is 98.8%. The probabilities exceed 90%, suggesting that the outcomes of the potential profile analysis in this study are relatively reliable. Refer to Table 3 for further details.

**Table 3:** Average attribution rates for three potential categories of perceived risk of recurrence in stroke patients (*n* = 198).

Models	C1	C2	C3
C1	0.980	0.016	0.004
C2	0.010	0.982	0.008
C3	0.010	0.002	0.988

### Potential categorical characteristics of relapse risk perception in stroke patients

**Figure 1:**
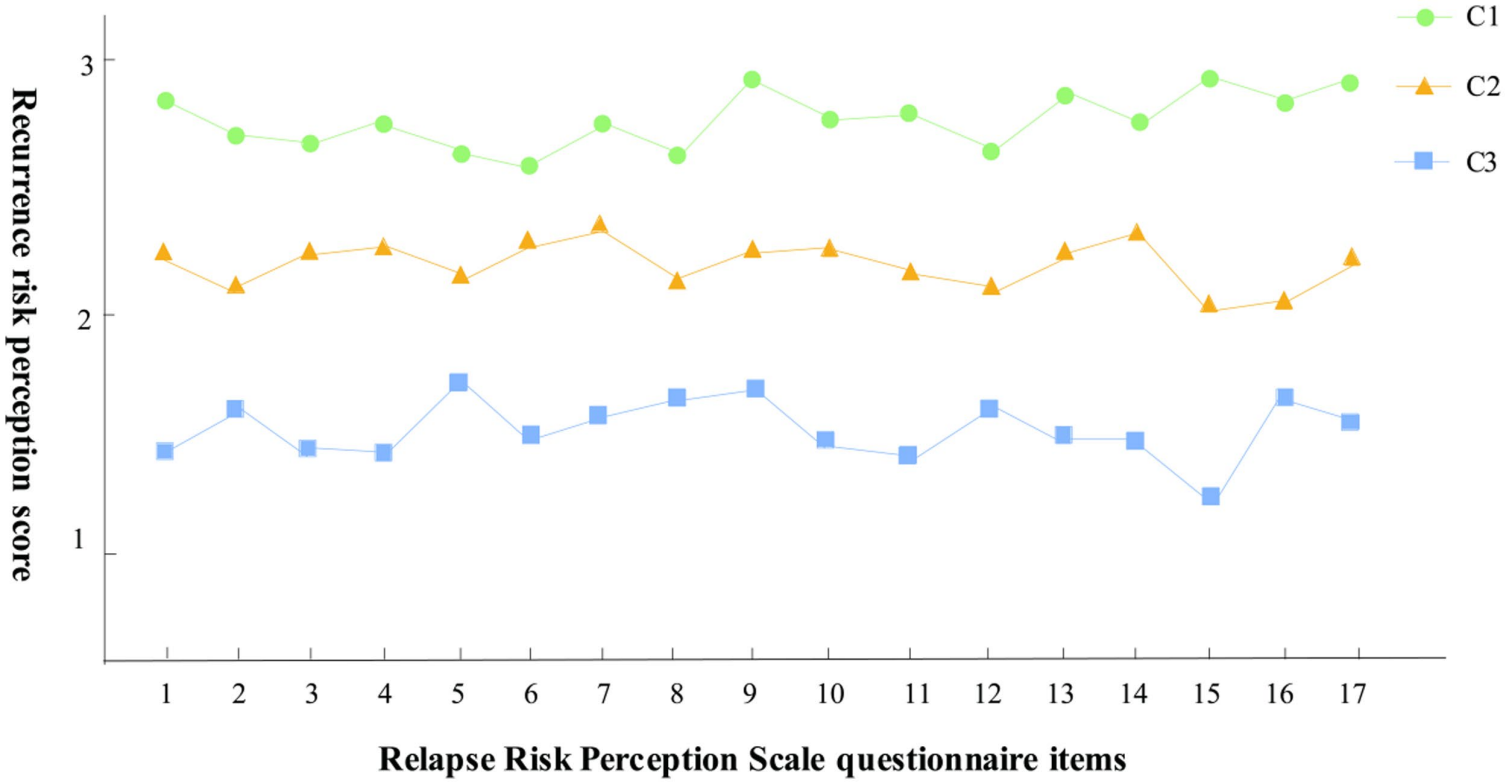
Numbers 1 to 17 in the figure represent specific items of the Relapse Risk Perception Scale questionnaire.

Results from potential profile analysis indicate that stroke patients can be classified into three distinct categories. The creation of line charts depicting the scores for each dimension of the relapse risk perception scale (refer to Figure 1) facilitates a clearer analysis of the characteristics of relapse risk perception among stroke patients across these three potential categories. Category 1 (C1) scored 2.66 ~ 2.94 points in the questionnaire of relapse risk perception category, which was always at a high level, so C1 was named “high level risk perception group,” and the proportion of C1 group was 35.8% (71 cases). Category 2 (C2) scored 21.93 ~ 2.25 points in the category of relapse risk perception questionnaire, maintaining the medium level, so C2 was named “medium level risk perception group,” and the proportion of C2 group was 43.3% (86 cases). Category 3 (C3) recurrence risk perception category questionnaire items score 1.25 ~ 1.66, maintained at a low level, so C3 was named “low level risk perception group,” C3 group accounted for 20.9% (41 cases).

### A univariate analysis of potential categories of relapse risk perception in stroke patients

The univariate analysis results indicated significant differences in age (*χ*^2^ = 11.132, *p* = 0.025), education background (*χ*^2^ = 5.523, *p* = 0.044), residence style (*χ*^2^ = 9.868, *p* = 0.007), duration of disease (*χ*^2^ = 13.142, *p* = 0.011), and stroke frequency (*χ*^2^ = 25.710, *P*<0.001), as presented in Table 4.

**Table 4:** General data of survey respondents and univariate analysis of their potential profile for recurrence risk perception (*n* = 198).

Variables	C1 (*n* = 71, %)	C2 (*n* = 86, %)	C3 (*n* = 41, %)	*χ*^2^	*p* value
Age (years)				11.132	0.025
<45	2 (2.81)	4 (4.65)	5 (12.20)		
45 ~ 59	26 (36.62)	35 (40.70)	23 (56.10)		
≥60	43 (60.57)	47 (54.65)	13 (31.70)		
Gender				2.293	0.318
Male	35 (49.30)	44 (51.16)	26 (63.41)		
Female	36 (50.70)	42 (48.84)	15 (36.59)		
Marital status				5.523	0.238
Unmarried	1 (1.41)	2 (2.33)	2 (4.88)		
Married	68 (95.77)	79 (91.86)	34 (82.93)		
Divorce	2 (2.82)	5 (5.81)	5 (12.19)		
Academic Qualifications				9.778	0.044
Junior high school or less	37 (52.11)	36 (41.86)	21 (51.22)		
High school	20 (28.17)	40 (46.51)	18 (43.90)		
Junior college and above	14 (19.72)	10 (11.63)	2 (4.88)		
Place of Residence				3.102	0.541
City	31 (43.66)	28 (32.56)	12 (29.27)		
Township	17 (23.94)	25 (29.07)	13 (31.71)		
Rural	23 (32.40)	33 (38.37)	16 (39.02)		
Lifestyle				9.868	0.007
Living alone	5 (7.04)	9 (10.47)	11 (26.83)		
Family Living	66 (92.96)	77 (89.53)	30 (73.17)		
Monthly household income (Yuan)				9.176	0.164
<4,000	26 (36.62)	42 (48.84)	23 (56.10)		
4,000 ~ 6,999	25 (35.21)	28 (332.56)	15 (36.59)		
7,000 ~ 10,000	15 (21.13)	10 (11.63)	2 (4.88)		
>10,000	5 (7.04)	6 (6.97)	1 (2.43)		
Hypertension				1.548	0.461
Yes	28 (39.44)	30 (34.88)	19 (46.34)		
No	43 (60.56)	56 (65.12)	22 (53.66)		
Coronary Heart Disease				0.808	0.668
Yes	23 (32.39)	25 (29.07)	10 (24.39)		
No	48 (67.61)	61 (70.93)	31 (75.61)		
Diabetes				2.547	0.280
Yes	15 (21.13)	20 (23.26)	14 (34.15)		
No	56 (78.87)	66 (76.74)	27 (65.85)		
Duration of disease (years)				13.142	0.011
<1	21 (29.58)	42 (48.84)	21 (51.22)		
1 ~ 3	23 (32.39)	29 (33.72)	14 (34.15)		
>3	27 (38.03)	15 (17.44)	6 (14.63)		
Strokes number				25.710	<0.001
1	42 (59.15)	74 (86.05)	39 (95.12)		
2	22 (30.99)	10 (11.63)	2 (4.88)		
≥3	7 (9.86)	2 (2.32)	0		
Type of insurance				4.581	0.101
Resident insurance	33 (46.48)	41 (47.67)	27 (65.85)		
Employee health insurance	38 (53.52)	45 (52.33)	14 (34.15)		

C1 “High Level risk Perception Group,” C2 “medium level risk Perception Group,” C3 “Low level risk Perception Group”.

### Multivariate analysis of three potential profiles regarding relapse risk perception in stroke patients

The recurrence risk perception score of stroke patients served as the dependent variable, while the indicators demonstrating statistically significant differences in the univariate analysis were utilized as independent variables for logistic regression analysis. The findings indicated that, using the C3 group as a reference, age (categorized as < 45 years old = 1, 45–59 years old = 2, ≥60 years old = 3, with < 45 years old as the control), educational background (junior high school and below = 1, senior high school = 2, junior college and above = 3, with junior high school and below as the control), living style (solitary = 1, family living = 2), disease duration (< 1 year = 1, 1–3 years = 2, > 3 years = 3, with < 1 year as the control), and the number of strokes (1 = 1, 2 = 2, ≥3 = 3, with 1 as the control) were identified as influencing factors for the perceived risk of recurrence, as detailed in Table 5.

**Table 5:** Multivariate analysis of three potential profiles of relapse risk perception in stroke patients (*n =* 198).

Item	*B*	*SE*	Walds *χ^2^*	*P*	*OR*	95% *CI*
Group C1 vs. Group C3						
Constant	2.050	0.425	13.163	<0.001	5.250	–
Age (years)						
45 ~ 59	−0.125	0.622	6.380	0.012	0.880	0.612 ~ 0.985
≥60	−0.454	0.556	4.913	0.027	0.631	0.534 ~ 0.840
Living style						
Family living	−0.202	0.091	4.977	0.026	0.812	0.720 ~ 0.907
Duration of disease (years)						
1 ~ 3	−0.572	0.241	5.597	0.018	0.561	0.347 ~ 0.902
>3	−0.909	0.387	5.491	0.019	0.404	0.263 ~ 0.693
Stroke frequency						
2	−0.862	0.358	5.807	0.016	0.420	0.208 ~ 0.850
≥3	−0.913	0.350	6.791	0.009	0.401	0.202 ~ 0.797
Group C2 vs. Group C3						
Constant	1.152	0.224	27.525	<0.001	3.121	–
Age (years)						
60 ~ 70	−0.264	0.344	6.373	0.012	0.759	0.530 ~ 0.921
>70	−0.501	0.501	7.300	0.003	0.602	0.534 ~ 0.855
Living style						
Family living	−0.153	0.050	8.554	0.004	0.851	0.645 ~ 0.964
Duration of disease (years)						
1 ~ 3	−0.064	0.025	6.752	0.009	0.934	0.862 ~ 0.989
>3	−0.136	0.056	5.695	0.017	0.869	0.756 ~ 0.915
Stroke frequency						
2	−0.626	0.243	6.531	0.011	0.534	0.330 ~ 0.862
≥3	−0.651	0.306	4.513	0.034	0.523	0.284 ~ 0.950

C1 “High level risk perception Group,” C2 “medium level risk perception Group,” C3 “Low level risk perception group”.

### Analysis of differences in self-management ability of stroke patients with different profiles of recurrence risk perception

The self-management ability score of 198 stroke patients was (85.69 ± 12.33). The self-management ability scores of stroke patients categorized by varying recurrence risk perception profiles were analyzed and compared. The findings indicated that the self-management ability of patients in the high-level risk perception group exceeded that of both the medium-level (*p* < 0.01) and low-level risk perception groups (*p* < 0.01). Additionally, the medium-level risk perception group demonstrated a higher self-management ability than the low-level risk perception group (*p* < 0.01), as illustrated in Table 6.

**Table 6:** Analysis of the difference in self-management ability of stroke patients with different recurrence risk perception profiles.

Variable	C1	C2	C3	*F*	Compare the results in pairs
Communicate the dimensions of assertiveness	17.06 ± 4.94	15.34 ± 4.54	12.03 ± 3.08	12.362	C1 > C3^**^, C2 > C3^**^, C1 > C2
Dimensions of perceived competence for professional guidance	12.18 ± 3.55	12.06 ± 3.33	9.26 ± 2.11	6.326	C1 > C3^**^, C2 > C3
Dimensions of self-management decision-making ability	16.64 ± 4.97	15.38 ± 3.95	11.68 ± 2.49	8.529	C1 > C3^**^, C2 > C3^**^, C1 > C2
Stroke impact dimensions	16.52 ± 4.15	15.65 ± 4.16	12.26 ± 2.31	6.422	C1 > C3^**^, C2 > C3
Perceive the assertiveness competence dimension	16.94 ± 4.37	14.99 ± 4.05	12.14 ± 2.99	8.997	C1 > C3^**^, C2 > C3^**^, C1 > C2
Health management ability dimension	16.21 ± 4.53	14.66 ± 4.11	11.52 ± 2.85	11.033	C1 > C3^**^, C2 > C3^**^, C1 > C2
Overall score for self-management ability	95.55 ± 12.33	85.08 ± 11.53	69.89 ± 9.54	35.966	C1 > C3^**^, C1 > C2^**^, C2 > C3

C1 “high level risk perception group,” C2 “medium level risk perception group,” C3 “low level risk perception group,” * means *p* < 0.05, ** means *p* < 0.01.

## Discussion

### Relapse risk perception in stroke patients is at a medium-high level

The results of this study indicated that stroke patients’ perception of recurrence risk was at a medium-high level. This aligns with the findings (19, 20) of prior studies. The majority of patients in this study were middle-aged and older adult individuals who demonstrated increased attention to their health care and heightened awareness of their conditions post-illness. This observation accounts for their medium-high level of relapse perception. Wang et al. (20) confirmed that patients with a heightened perception of recurrence risk exhibited a greater willingness to recover in later stages, increased enthusiasm for exercise and compliance, and a comparatively improved prognosis through effective rehabilitation exercises. This indicates that clinical staff ought to perform prompt and appropriate evaluations of stroke patients’ perceptions regarding recurrence risk, and implement necessary health management for those with low recurrence risk perception scores to mitigate the likelihood of stroke recurrence.

### The recurrence risk perception of stroke patients has 3 different profile categories

In this study, through LPA, it was found that the recurrence risk perception of stroke patients had 3 potential profile categories, namely, the high level risk perception, medium level risk perception, and low level risk perception. This finding highlights the heterogeneity in the recurrence risk perception status of stroke patients. Most domestic and foreign studies on the perception of recurrence risk among stroke patients concentrate on assessing the current state of the disease and analyzing influencing factors (21). This study analyzes the internal recurrence risk perception among stroke patients, categorizing their ability to perceive recurrence risk into distinct groups to facilitate targeted interventions. 35.8% of patients belonged to the high-risk perception group. Despite their disease challenges, these patients demonstrated a significant capacity for recognition and were able to identify and respond positively, potentially correlating with favorable prognostic outcomes. 43.3% of patients fell into the medium-level risk perception group, suggesting that a majority of stroke patients possessed a moderate ability to perceive the risk of recurrence. These patients demonstrated some ability to perceive recurrence risk; however, their recognition capacity was inferior to that of the high-level group. Lin et al. (16), in a 10-year cohort study, reported stroke recurrence rates of 7.7% at 3 months, 9.5% at 6 months, and 16.1% at 1 year. Their findings suggest that enhancing the perception levels of patients in the medium-level perception group significantly contributes to reducing the recurrence rate. 20.9% of patients belonged to the low-risk perception group. The patients in this group exhibited insufficient attention to the disease, resulting in low treatment enthusiasm and compliance, thereby significantly increasing the likelihood of recurrence. This cohort of patients required diligent oversight and management by healthcare professionals.

### Analysis of factors influencing relapse risk perception among stroke patients categorized into three distinct profiles

This study’s results indicated that the perception of recurrence risk among stroke patients varied significantly based on age, living arrangements, disease duration, and stroke frequency. Previous studies indicate (22) that older patients exhibit a higher level of risk perception compared to middle-aged and young adults. Older adult patients, in contrast to young and middle-aged individuals, exhibit a greater decline in physical function, leading to increased attention to their health conditions, enhanced recognition of abnormal symptoms, and heightened awareness of relapse. The impact of residence style on perceived relapse risk may be evident in the recognition initiative. Patients living alone exhibit weak social relationships, an inadequate support system post-illness, and a lack of confidence and enthusiasm in treatment. This leads to insufficient perceptual motivation and, consequently, a (23) low perceived level of relapse. Patients with a prolonged disease course tend to have a greater awareness of the associated complications. Over time, their understanding of the disease deepens, leading to an enhanced perception of relapse due to increased access to disease-related information (24). Individuals with a greater number of strokes exhibited an elevated level of perception. Following an initial stroke, patients experience a degree of uncertainty regarding disease progression; however, they often neglect to adequately address the condition, leading to insufficient awareness of recurrence and diminished risk perception capabilities.

### The relationship between the perception of recurrence risk and self-management capabilities in stroke patients

This study indicates that stroke patients exhibit a low level of self-management ability. Furthermore, patients categorized in the high-level risk perception group demonstrated a higher self-management ability compared to those in the medium-level risk perception group, who, in turn, outperformed the low-level risk perception group. This indicates that the ability to perceive relapse risk is crucial for self-management capabilities. Patients with a heightened awareness of their relapse risks are likely to recognize their vulnerabilities sooner (25), allowing them to modify their behaviors, such as refraining from detrimental habits and enhancing adherence to medical recommendations, thereby improving their self-management skills. This indicates that clinical staff should incorporate the assessment and intervention of recurrence risk perception into the nursing plan. Middle-aged and young patients, those living alone, individuals with a disease duration of less than one year, and first-time stroke patients should be monitored for low risk perception levels. Timely interventions and adjustments are necessary to enhance their awareness of recurrence risk. The follow-up treatment and prognosis of stroke patients are of considerable importance.

### Implications for clinical practice

This gives the clinical work a certain inspiration. First, medical personnel can conduct stratified risk assessment and education: standardized tools are used to comprehensively assess the level of risk perception of stroke patients at admission, and they are classified into three grades: high, medium, and low. Individualized health education programs are designed according to different risk perception levels. For patients in the low level risk perception group, the harm of stroke and the importance of self-management were emphasized, and their risk perception was improved through case explanation and dissemination of popular science materials. For patients with moderate risk perception, specific self-management methods were further provided, such as diet, exercise, medication guidance, etc. For patients with high level of risk perception, more in-depth and professional health management knowledge, such as rehabilitation training skills, psychological adjustment methods, etc.

Secondly, adhere to personalized support and counseling: for different grades of patients, carry out targeted psychological counseling. Patients in the low level risk perception group may not pay enough attention to the disease, so they should be guided to pay attention to their own health. Patients in the moderate risk perception group may have some anxiety, which helps them face the disease correctly; Patients with high level of risk perception may have excessive worry, and psychological counseling is needed to enhance their confidence.

Finally, continuous follow-up and reinforcement: Establish a long-term follow-up mechanism, dynamically adjust intervention programs according to patients’ self-management, and continuously strengthen education and support to help patients gradually improve their self-management ability.

### Limitations of this study

There are some limitations to this study. The single-center survey was conducted in the form of self-report, and there may be potential biases in cognitive and social expectations when collecting and analyzing individual health behavior data, which may have some impact on the study results. Additionally, there may be omissions in the factors influencing relapse risk perception ability. In the later stage, the sample size will be increased, and the analysis of influencing factors will be enhanced to more effectively identify stroke patients with varying profiles of relapse risk perception. Additionally, relevant health management strategies will be formulated to mitigate their relapse risk.

### Future research direction

Later, a longitudinal survey will be conducted to track the changes in risk perception and self-management ability of the same group at different points in time, so as to clarify the causal relationship between the two and solve the limitations of cross-sectional design. In addition, analysis of influencing factors and qualitative interviews will be conducted for self-behavior management of stroke patients in the later stage.

## Summary

This study employed potential profile analysis to categorize the recurrence risk perception of stroke patients into three distinct groups: high, medium, and low risk perception levels. Age, residence style, duration of disease, and stroke frequency influenced the three potential profile categories. The perceived recurrence risk associated with various profiles was closely linked to patients’ self-management capabilities. Clinical staff must closely monitor patients categorized within the low -level risk perception group and implement strategies to enhance their understanding of recurrence risk. This approach positively influences patients’ self-management capabilities, aids in recurrence prevention, and enhances overall prognosis.

## Data Availability

The original contributions presented in the study are included in the article/supplementary material, further inquiries can be directed to the corresponding author.
